# Supplementary figures and images for: Mitochondrial respiration contributes to the interferon gamma response in antigen-presenting cells
Source: eLife. 2021 Nov 2;10:e65109. doi: 10.7554/eLife.65109 (PMC8598164; doi:10.7554/eLife.65109)

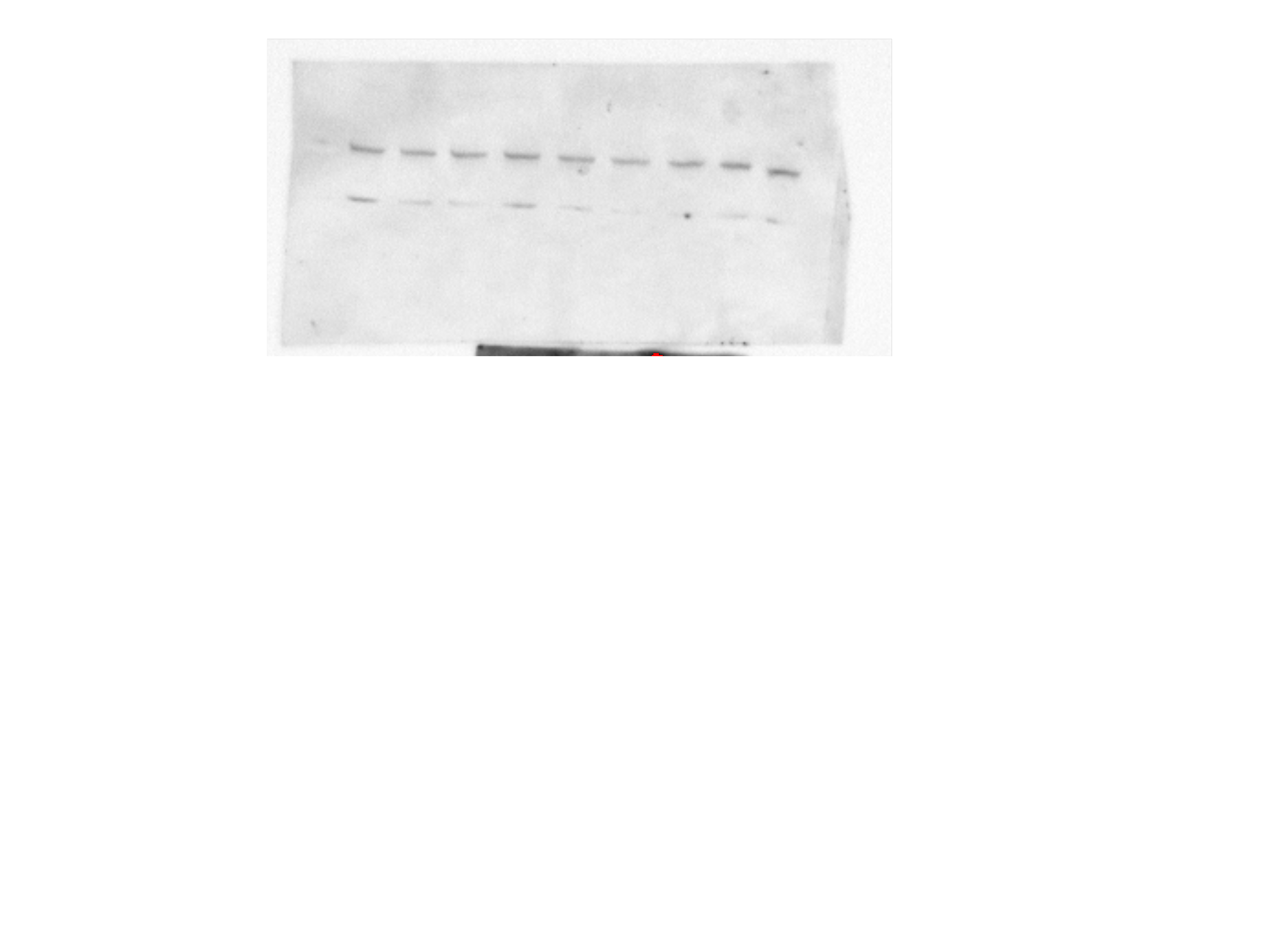

Supplement: Figure 6—source data 1. [file elife-65109-fig6-data1.zip › Figure6_SourceData1/Stat1total_raw.tiff]

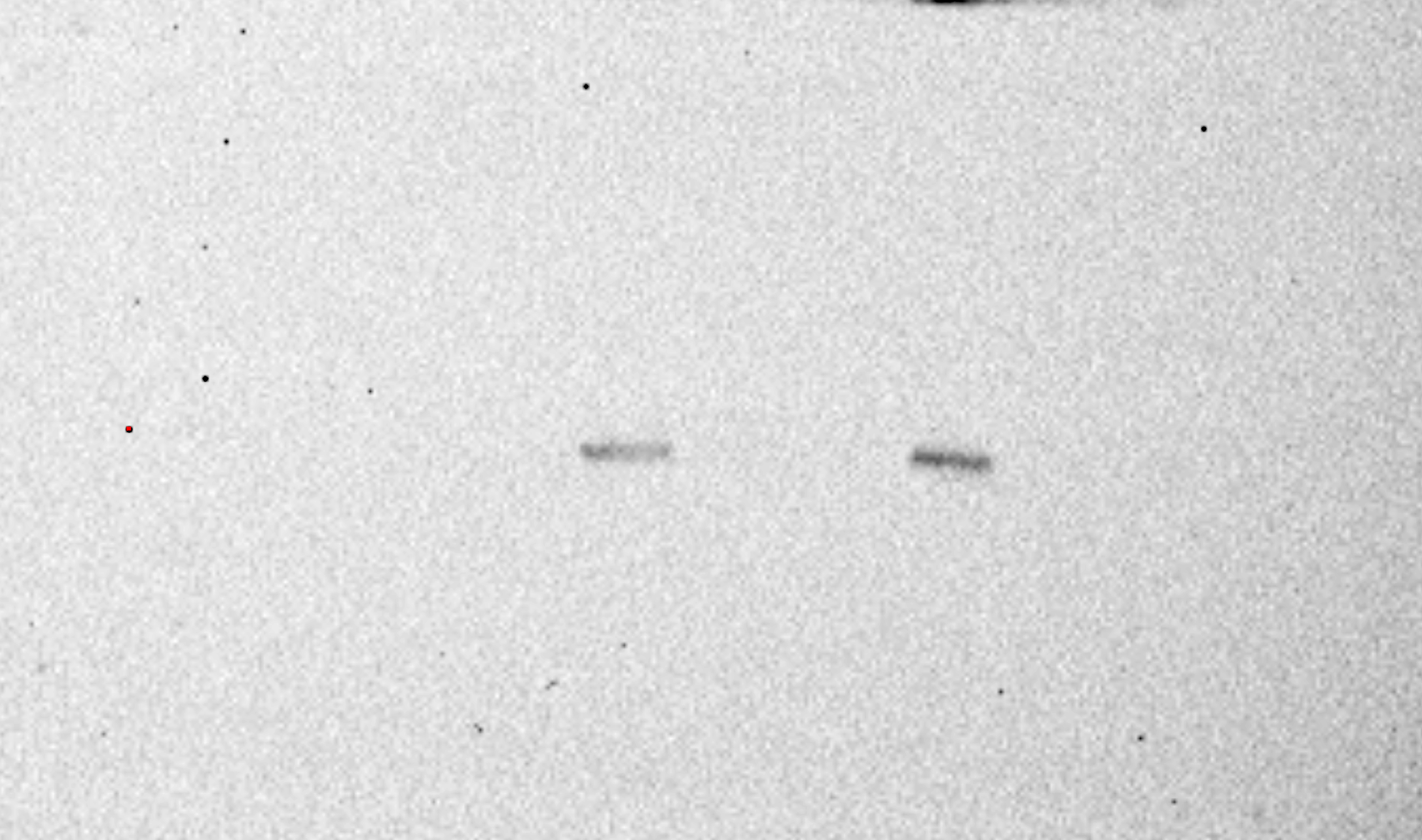

Supplement: Figure 6—source data 1. [file elife-65109-fig6-data1.zip › Figure6_SourceData1/pJak2_raw.tiff]

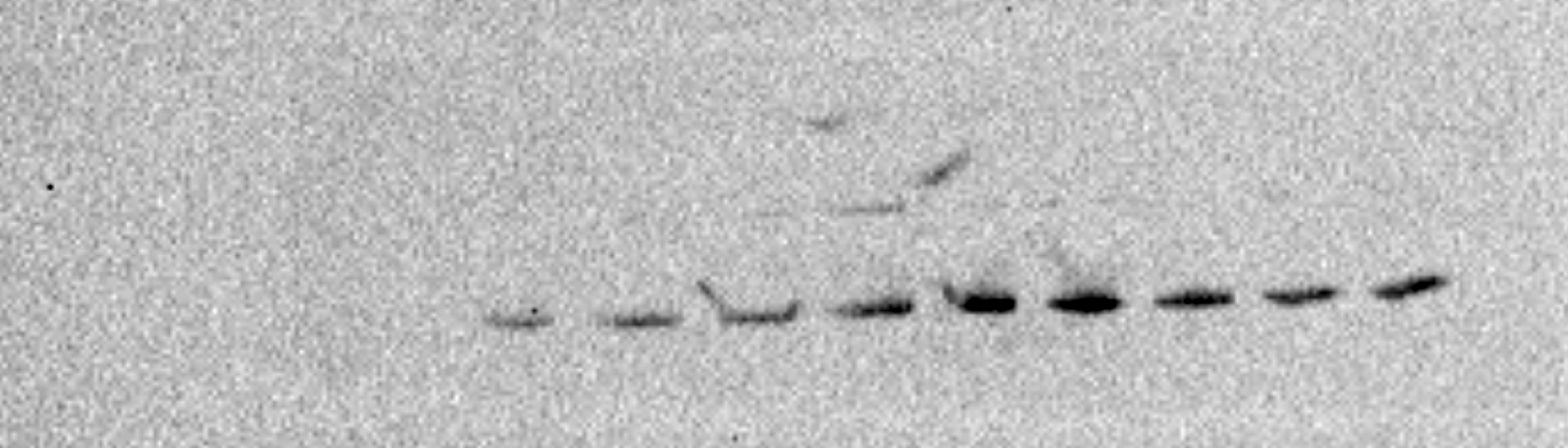

Supplement: Figure 6—source data 1. [file elife-65109-fig6-data1.zip › Figure6_SourceData1/Jak2Total_Raw.tiff]

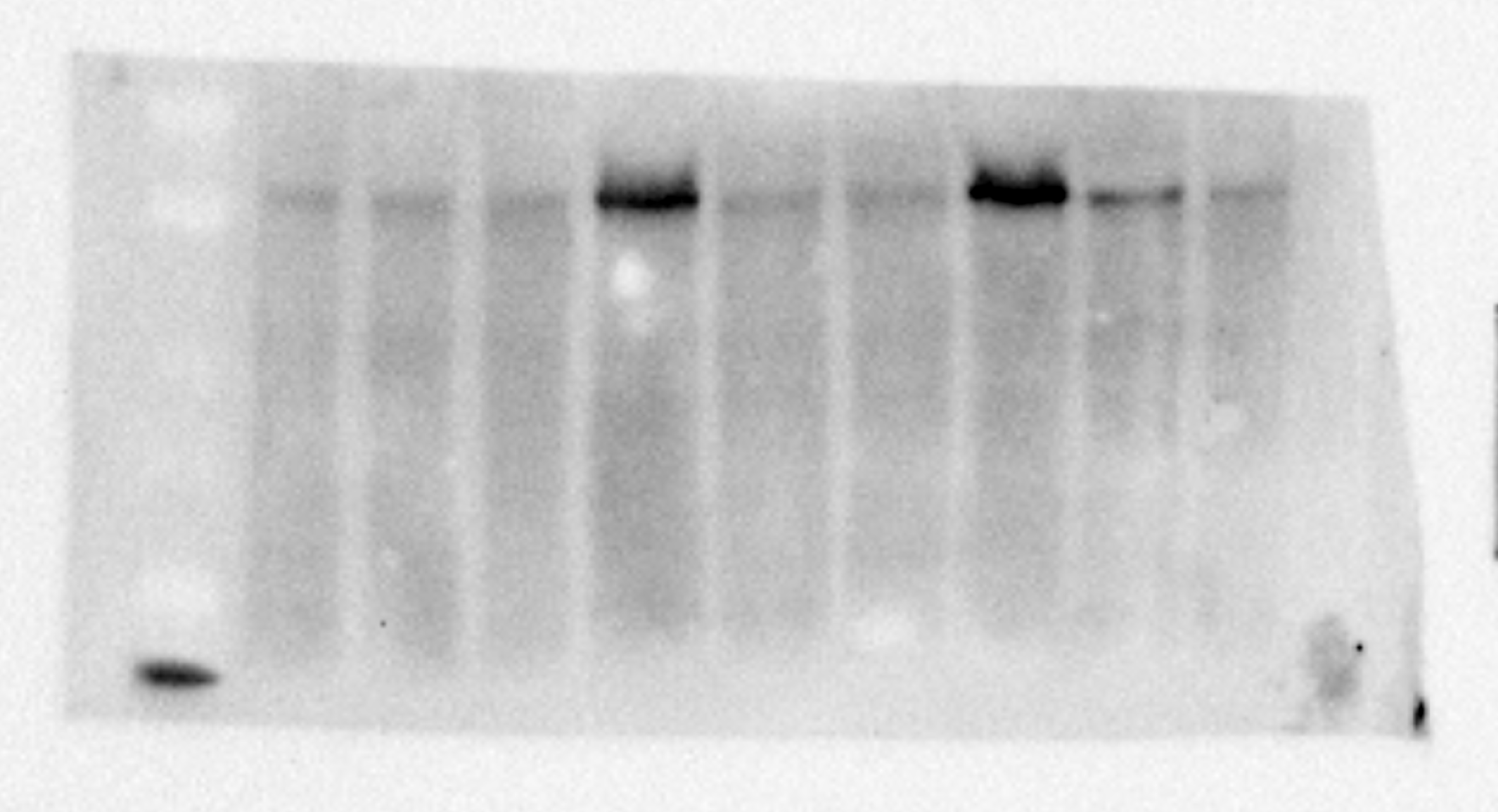

Supplement: Figure 6—source data 1. [file elife-65109-fig6-data1.zip › Figure6_SourceData1/pStat1_Ser_Raw.tiff]

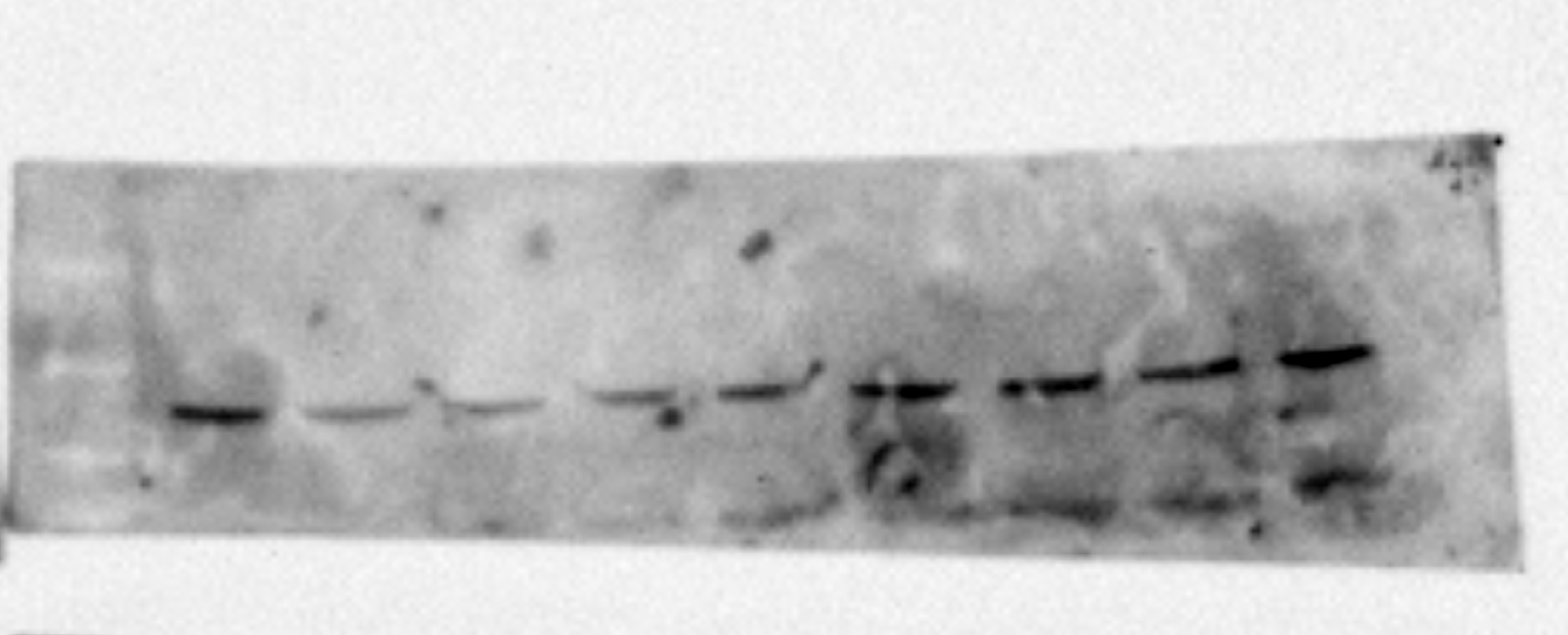

Supplement: Figure 6—source data 1. [file elife-65109-fig6-data1.zip › Figure6_SourceData1/ifngr_raw.tiff]

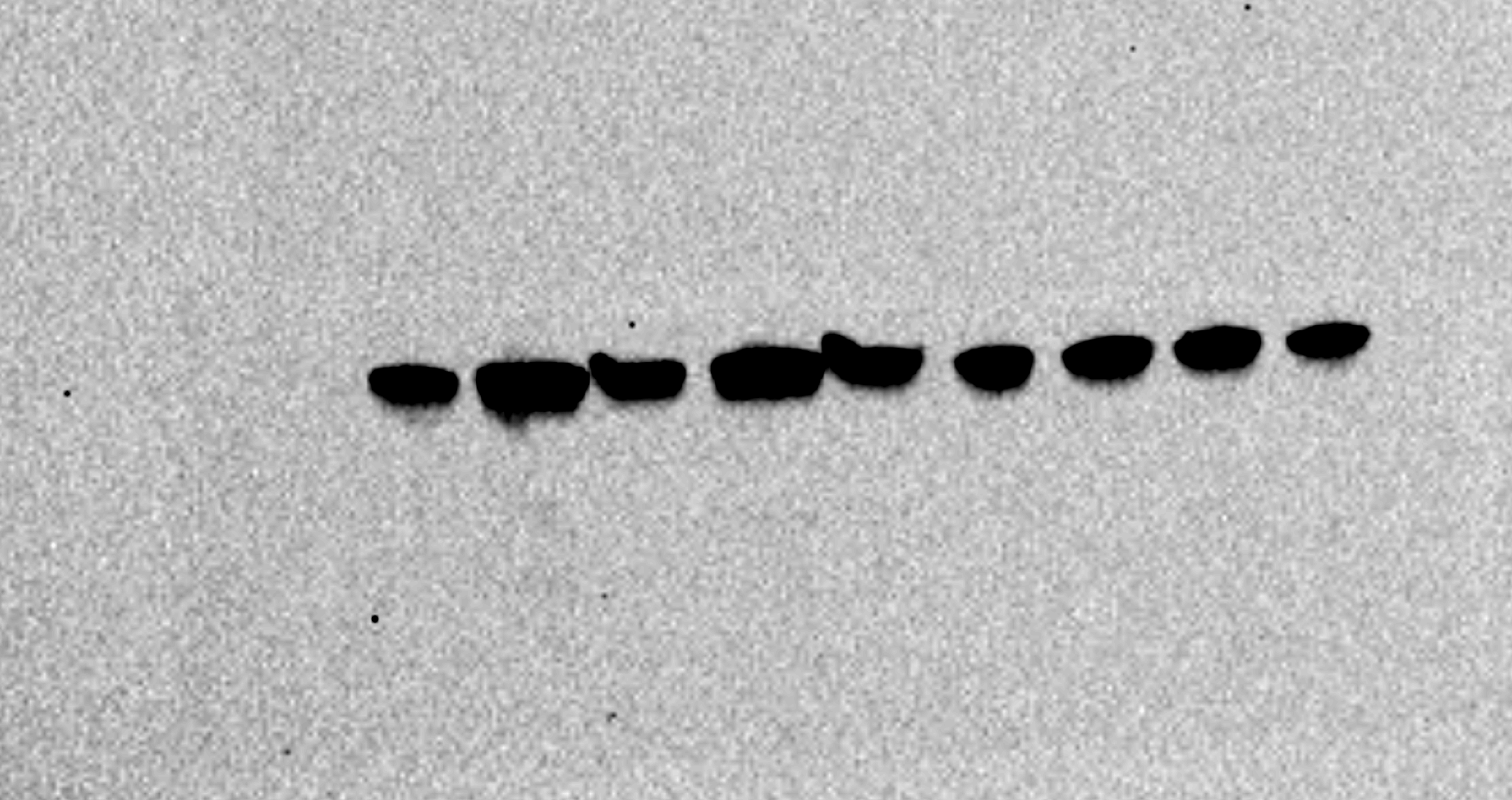

Supplement: Figure 6—source data 1. [file elife-65109-fig6-data1.zip › Figure6_SourceData1/Actin_Raw.tiff]

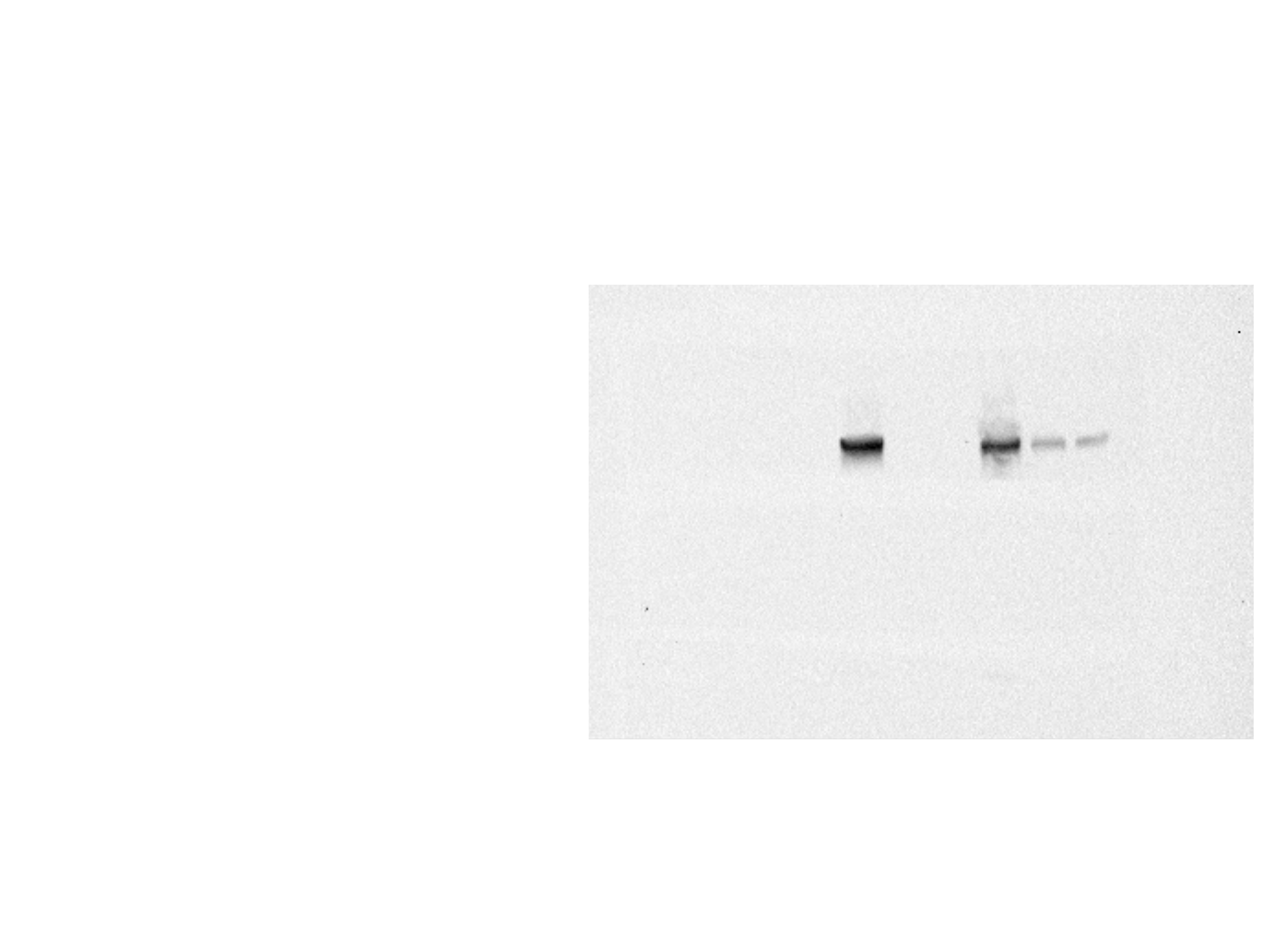

Supplement: Figure 6—source data 1. [file elife-65109-fig6-data1.zip › Figure6_SourceData1/pstat1_Tyr.tiff]

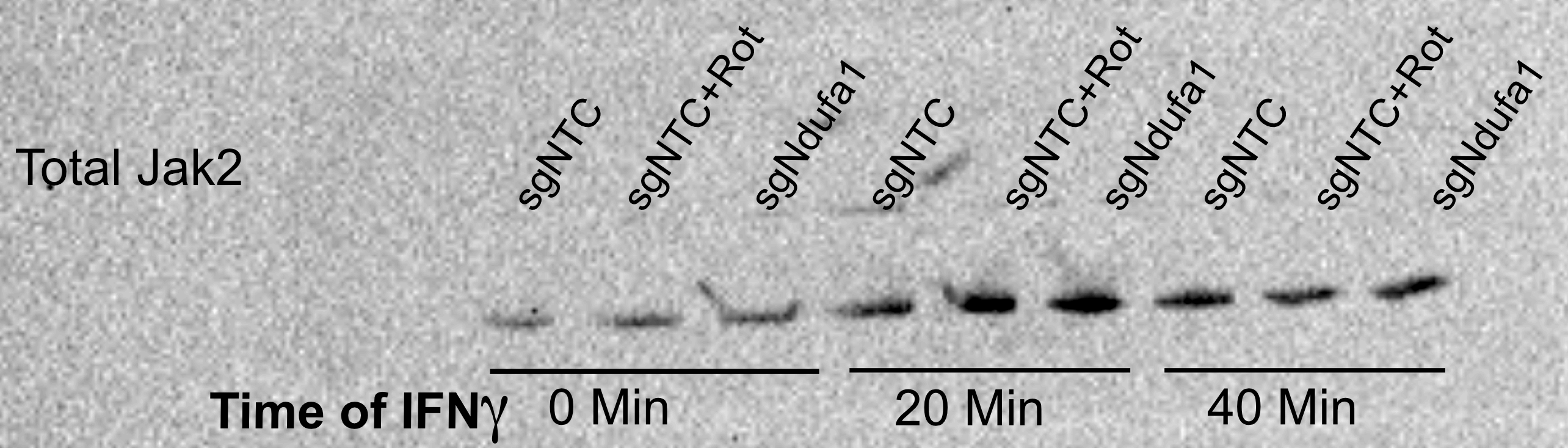

Supplement: Figure 6—source data 2. [file elife-65109-fig6-data2.zip › Figure6_SourceData2/Jak2Total_Labeled.tiff]

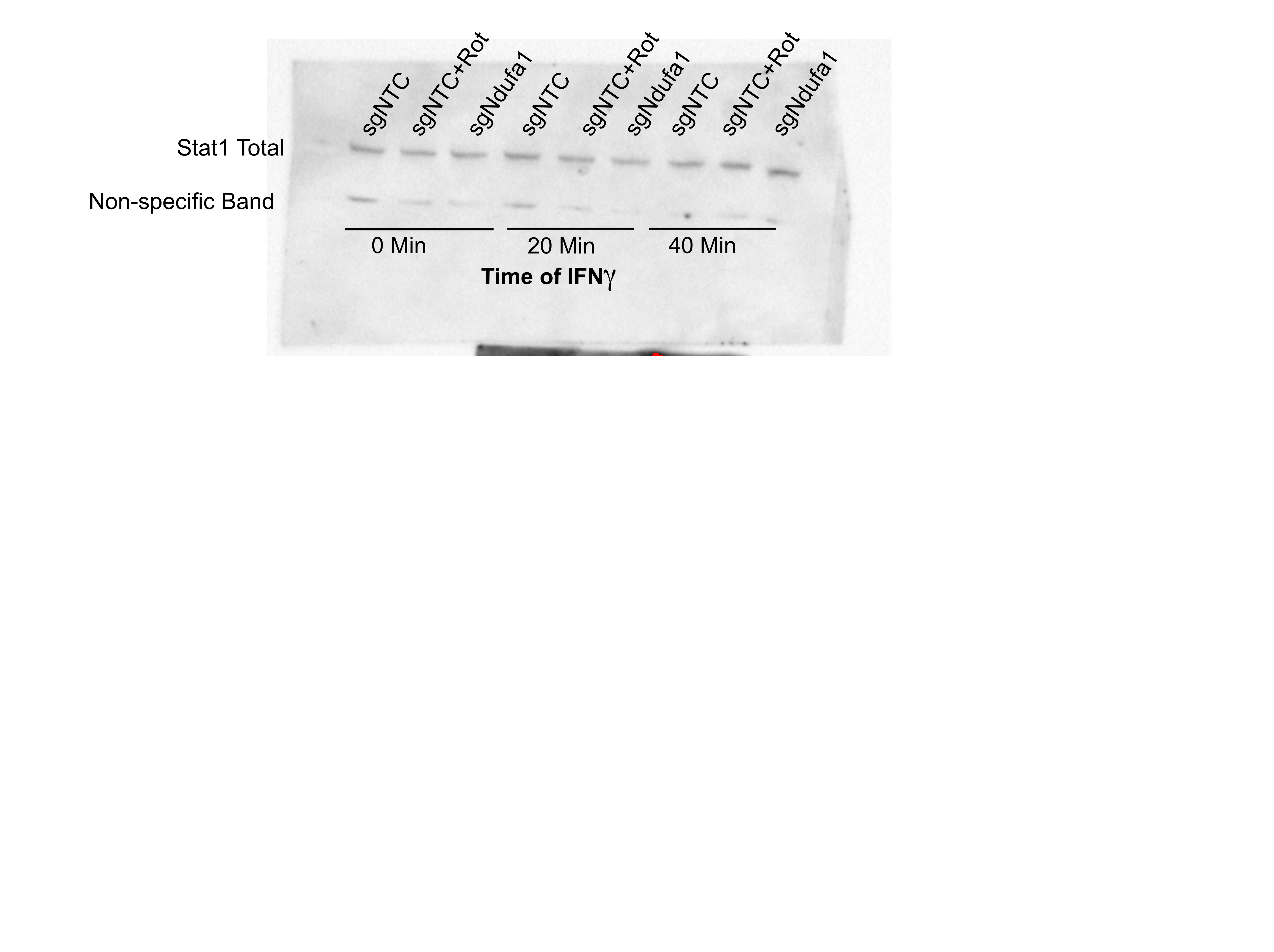

Supplement: Figure 6—source data 2. [file elife-65109-fig6-data2.zip › Figure6_SourceData2/Stat1total_labeled.tiff]

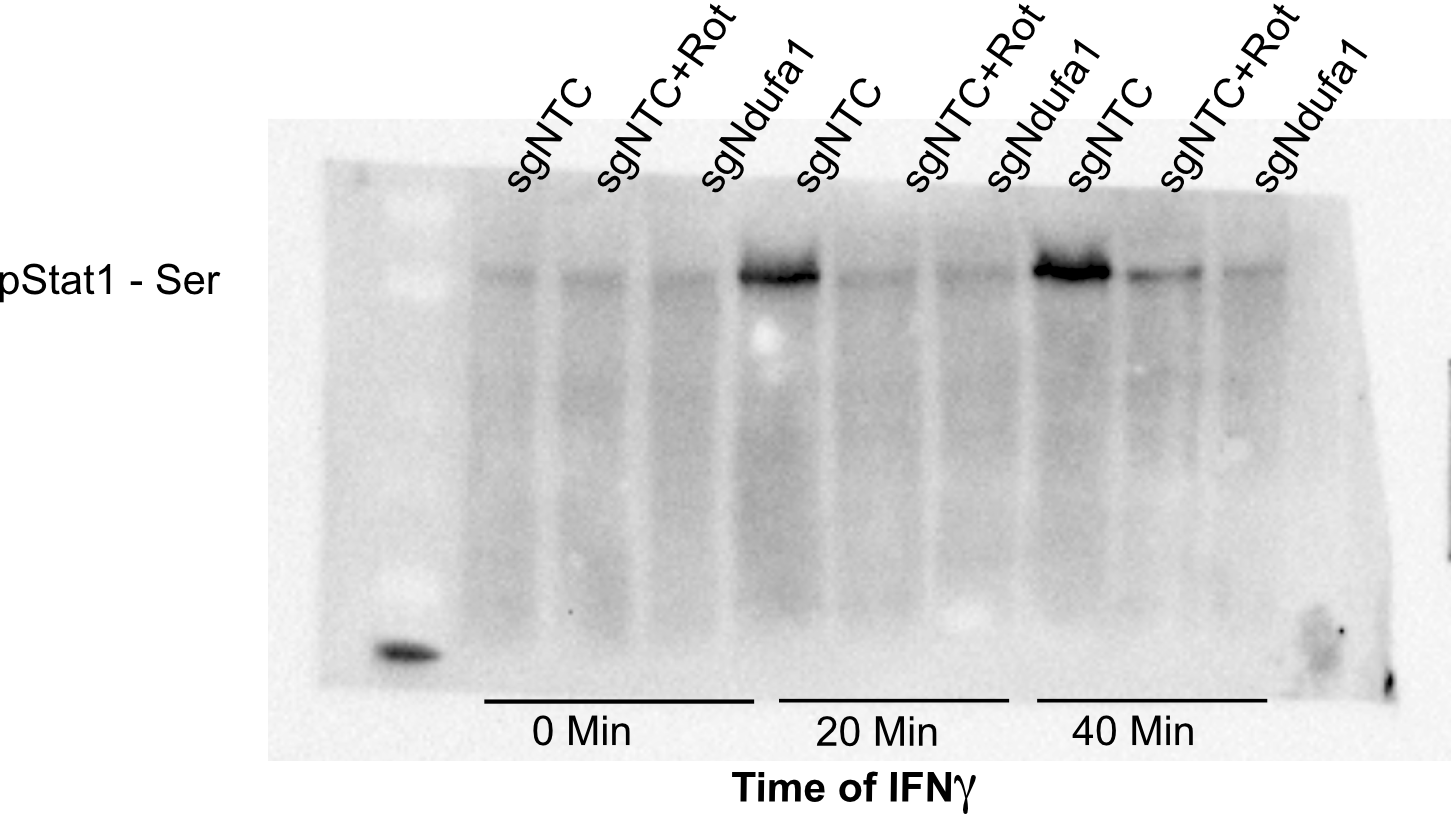

Supplement: Figure 6—source data 2. [file elife-65109-fig6-data2.zip › Figure6_SourceData2/pstat1_Ser_Labeled.tiff]

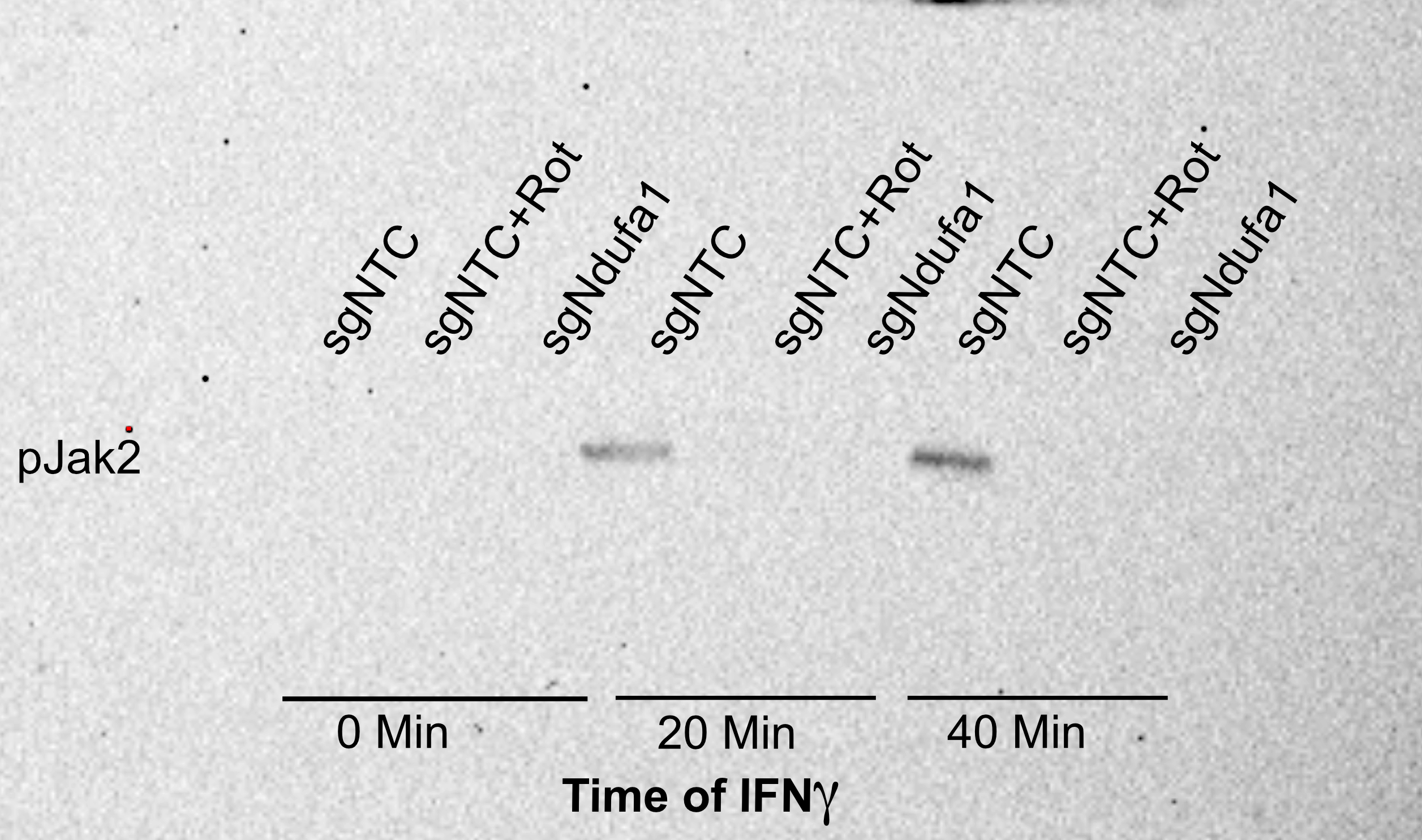

Supplement: Figure 6—source data 2. [file elife-65109-fig6-data2.zip › Figure6_SourceData2/pJak2_labeled.tiff]

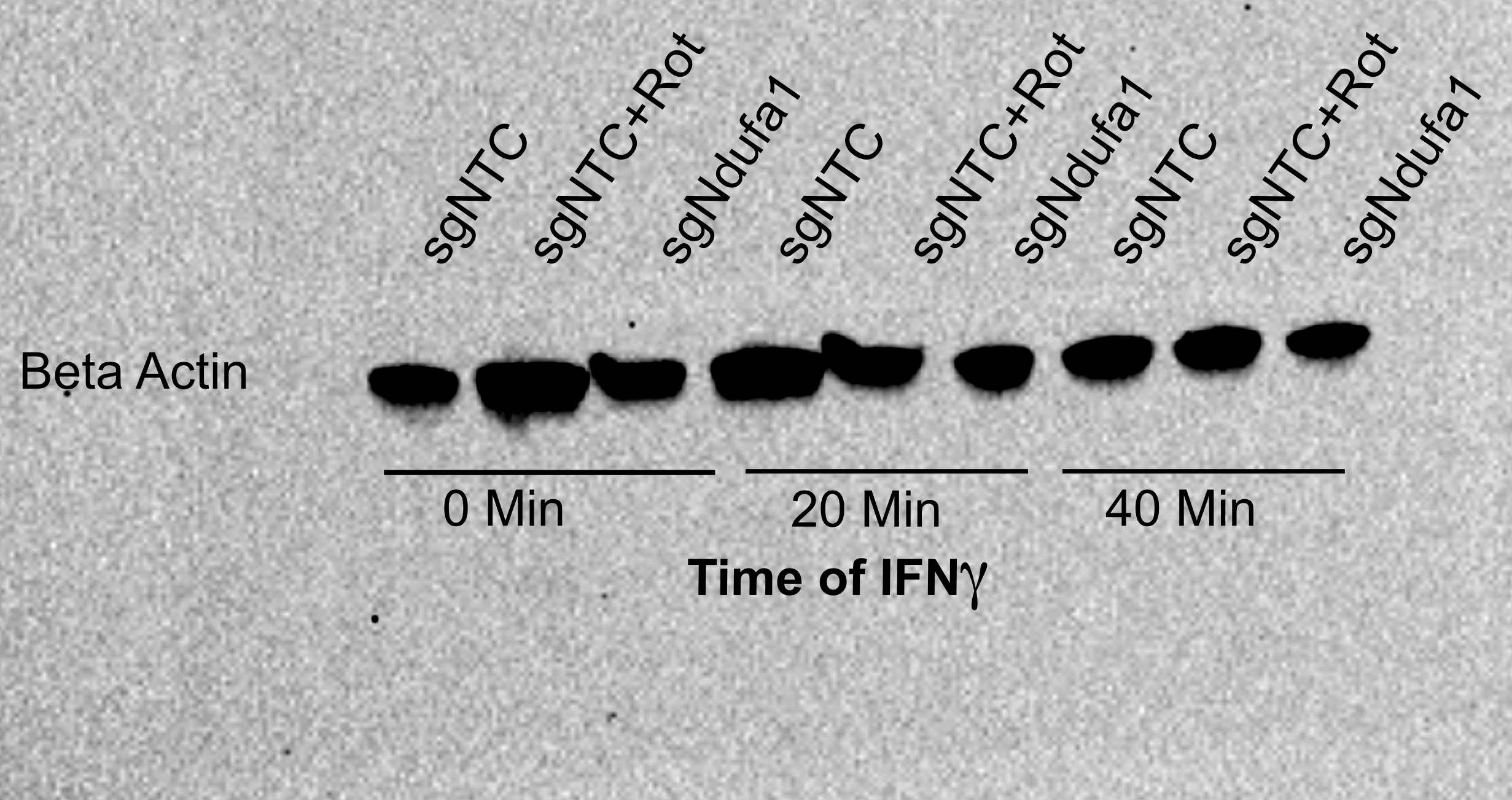

Supplement: Figure 6—source data 2. [file elife-65109-fig6-data2.zip › Figure6_SourceData2/Actin_Labeled.tiff]

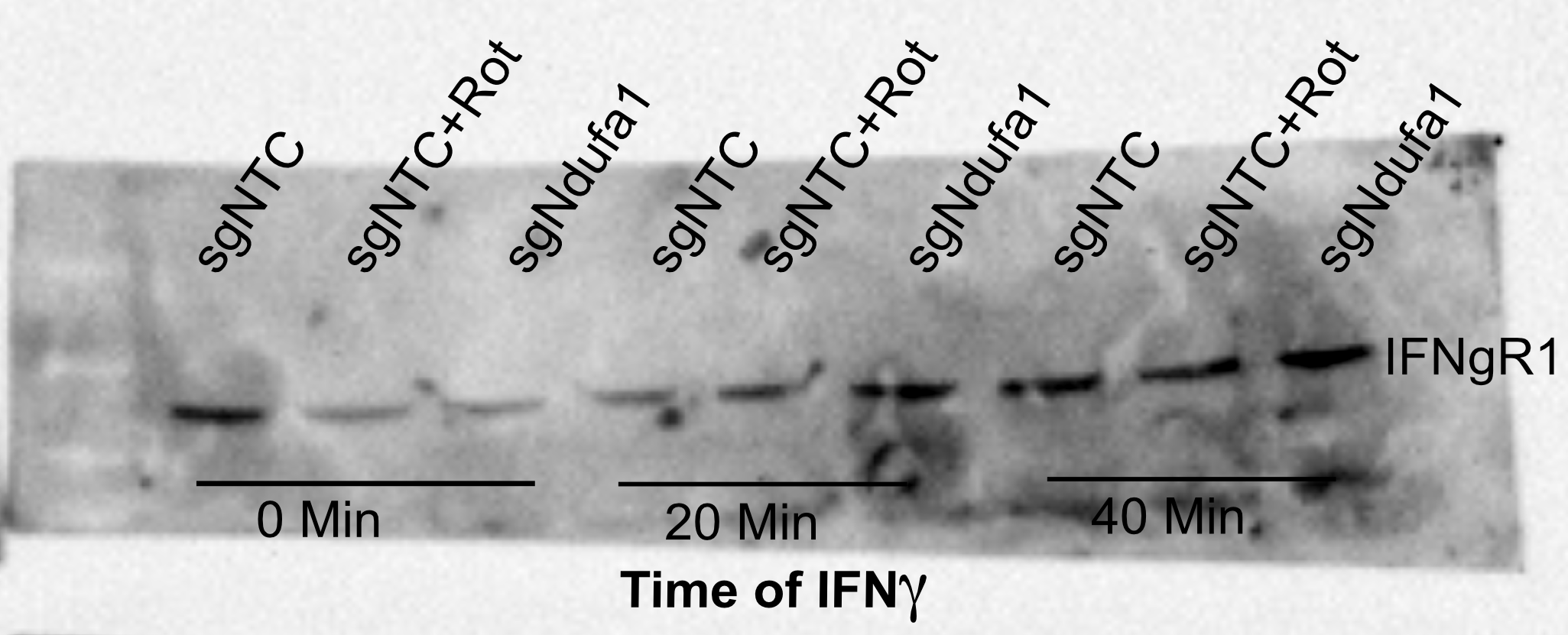

Supplement: Figure 6—source data 2. [file elife-65109-fig6-data2.zip › Figure6_SourceData2/ifngr_labeled.tiff]

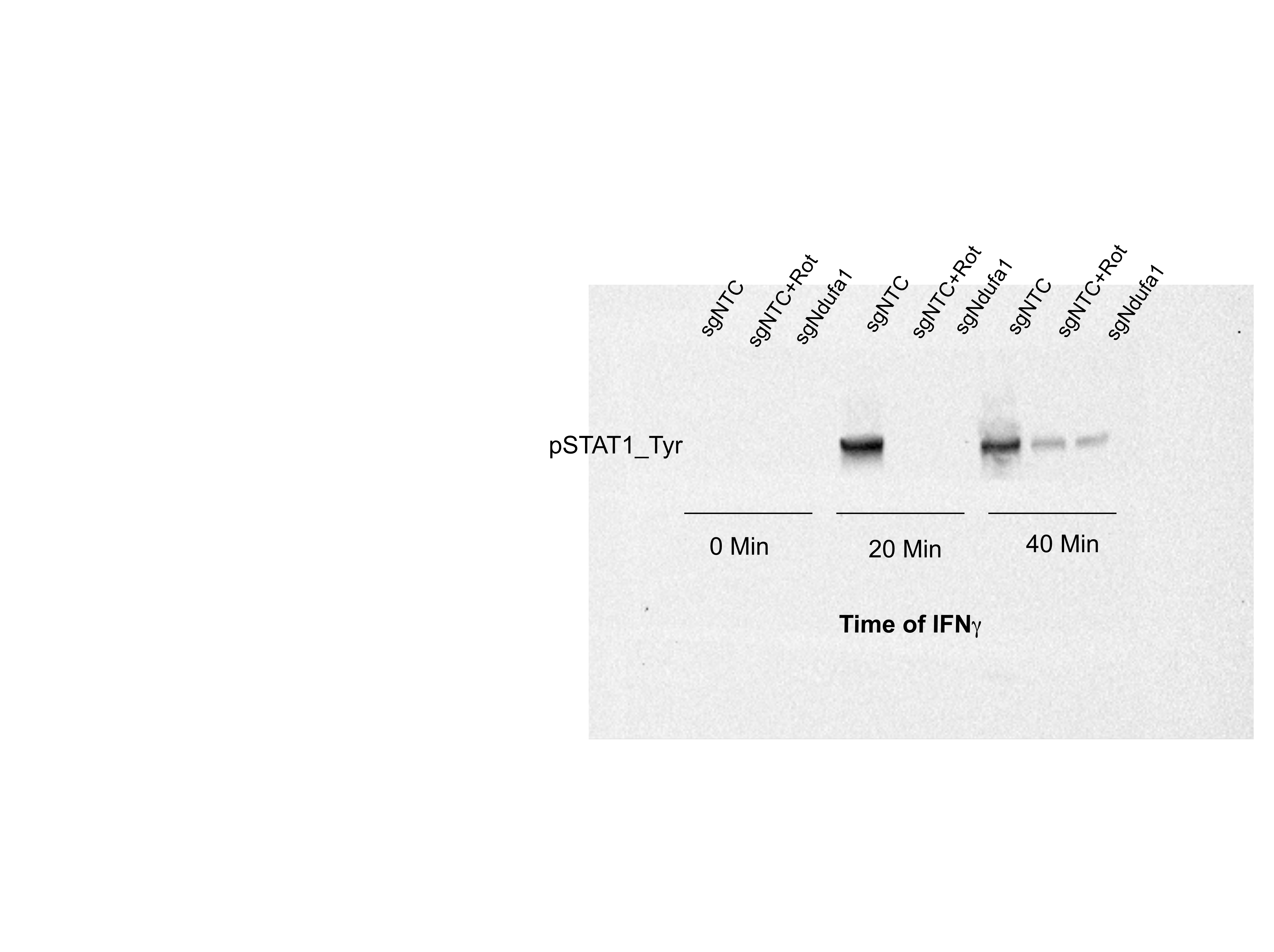

Supplement: Figure 6—source data 2. [file elife-65109-fig6-data2.zip › Figure6_SourceData2/pstat1_Tyr_labeled.tiff]
